# Proteomics Comparison of Cerebrospinal Fluid of Relapsing Remitting and Primary Progressive Multiple Sclerosis

**DOI:** 10.1371/journal.pone.0012442

**Published:** 2010-08-27

**Authors:** Marcel P. Stoop, Vaibhav Singh, Lennard J. Dekker, Mark K. Titulaer, Christoph Stingl, Peter C. Burgers, Peter A. E. Sillevis Smitt, Rogier Q. Hintzen, Theo M. Luider

**Affiliations:** Laboratories of Neuro-Oncology/Clinical and Cancer Proteomics, Department of Neurology, Erasmus University Medical Centre, Rotterdam, The Netherlands; University of North Dakota, United States of America

## Abstract

**Background:**

Based on clinical representation of disease symptoms multiple sclerosis (MScl) patients can be divided into two major subtypes; relapsing remitting (RR) MScl (85–90%) and primary progressive (PP) MScl (10–15%). Proteomics analysis of cerebrospinal fluid (CSF) has detected a number of proteins that were elevated in MScl patients. Here we specifically aimed to differentiate between the PP and RR subtypes of MScl by comparing CSF proteins.

**Methodology/Principal Findings:**

CSF samples (n = 31) were handled according to the same protocol for quantitative mass spectrometry measurements we reported previously. In the comparison of PP MScl versus RR MScl we observed a number of differentially abundant proteins, such as protein jagged-1 and vitamin D-binding protein. Protein jagged-1 was over three times less abundant in PP MScl compared to RR MScl. Vitamin D-binding protein was only detected in the RR MScl samples. These two proteins were validated by independent techniques (western blot and ELISA) as differentially abundant in the comparison between both MScl types.

**Conclusions/Significance:**

The main finding of this comparative study is the observation that the proteome profiles of CSF in PP and RR MScl patients overlap to a large extent. Still, a number of differences could be observed. Protein jagged-1 is a ligand for multiple Notch receptors and involved in the mediation of Notch signaling. It is suggested in literature that the Notch pathway is involved in the remyelination of MScl lesions. Aberration of normal homeostasis of Vitamin D, of which approximately 90% is bound to vitamin D-binding protein, has been widely implicated in MScl for some years now. Vitamin D directly and indirectly regulates the differentiation, activation of CD4+ T-lymphocytes and can prevent the development of autoimmune processes, and so it may be involved in neuroprotective elements in MScl.

## Introduction

Multiple sclerosis (MScl) can be divided into two major subtypes based on clinical representation of disease symptoms in the patients [1]. Between 85–90% of patients can be classified as having the relapsing remitting (RR) MScl subtype, in which disease relapses are followed by periods of remission, and 10–15% of all MScl patients are diagnosed with the primary progressive (PP) subtype [2]. Even within a single large Dutch MScl pedigree of 26 patients with similar genetic background, the percentage of patients with a PP phenotype remained 15% [3].

By definition, in PP patients disease progression is characterized by a progressive course without relapses or remissions from the onset of the disease [4]. PP patients tend to have lower inflammatory lesional activity, for which no immunological or genetic explanation has been identified yet. The scarce comparative neuropathological studies show a large overlap in lesional pathology, but indicate less inflammatory activity for PP, with still substantial axonal damage [5]. The general picture is that relapse onset and PP forms share substantial characteristics. In other words, it has remained a challenge to identify the biological parameters that determine a PP disease course.

Although proteomics analysis of active multiple sclerosis lesions may be a straightforward approach to study the processes involved in MScl disease pathways [6], this is very difficult to perform in living individuals. In most cases the pathology of the disease can only be investigated in post-mortem material, which quite frequently represents the end-stage of the disease. The study of CSF taken during disease appears a good alternative. CSF is in close contact with the CNS parenchyma and collects the products of the inflammatory and neurodegenerative processes of MScl activity.

Proteomics analysis of CSF has detected a number of proteins that were elevated in MScl patients [7], [8]. Additionally, differentially abundant proteins identified by proteomics, such as apolipoprotein A1 [9] and chromogranin A [10] were validated by other techniques. Other studies added additional data on elevated immunoglobulin expression in MScl CSF, as well as increased levels of apolipoprotein E [11], [12]. Yet in all currently reported proteomics CSF studies of MScl patients either only a single subtype of MScl patients or a combined group of all subtypes of MScl was studied, whilst the differences between the subtypes of MScl remained unexplored. Because RR MScl and PP MScl are very different in terms of disease course and disease progression, this also has therapeutic consequences. Hence, there are probably also differences on a biological and pathological level, which could, if determined, be very useful for elucidation of the biology and pathology of both disease types.

In the current study we specifically aimed to differentiate between the MScl patients and healthy controls and between both subtypes of MScl by comparing CSF proteins and peptides. Subsequently, the identified biomarker proteins and peptides were discussed in relation to the different pathological processes observed in RR MScl and PP MScl.

## Materials and Methods

### Ethics Statement

The Medical Ethical Committee, de commissie medisch ethische vraagstukken, of the Erasmus University Medical Centre in Rotterdam, The Netherlands, approved the study protocol and patients gave written consent. The approval numbers for this study are 200.721/2001/75 and 2006/188.

### Patient selection

The CSF samples of MScl patients, divided into two groups, RR MScl [13] and PP MScl [14], were collected from untreated patients undergoing routine diagnostic procedures by an experienced neurologist (RQH), and matched for presence or absence of oligoclonal bands. The healthy control CSF samples were taken from patients receiving spinal anesthesia prior to non-neurological minor surgical interventions, such as knee and hip replacements, groin rupture and Achilles tendon rupture. All samples were handled in exactly the same way after sampling, using a procedure that has been previously reported [12]. In brief, immediately after sampling, the CSF samples were centrifuged to discard cells and cellular elements and the total protein concentration and albumin concentrations were determined. The number of oligoclonal bands and the intrathecal cell count were also reported. The remaining volume of the samples was aliquoted and stored at −80°C, where they remained until sample preparation for this study. No extra freeze-thaw cycles were allowed.

### Sample preparation, measurement and analysis

The CSF samples were handled according to the same protocol for quantitative MALDI-FT-ICR MS measurements we reported previously [12], which consists of a blinded experiment in which the samples were digested by trypsin and subsequently measured by MALDI-FT-ICR (APEX IV Qe 9.6 Tesla MALDI-FT-ICR mass spectrometer (Bruker Daltonics, USA)). After calibration by means of omnipresent albumin peaks, an analysis matrix is generated. A univariate analysis, in which a p-value was determined for every peak position, was used for statistical analysis, in which two groups were compared at a time, for a total of three individual comparisons between the groups. The differentially abundant peaks (p<0.01) in the comparisons were considered for identification purposes. The fold increase of every identified peptide with p<0.01 in the comparisons was determined to confirm differential abundance between groups. Assessment of the statistical background, by means of permutation of a series of 50 scramblings of the samples for each comparison, was used to define a cut-off number for the determination of statistically significant differences between groups. In this permutation procedure all samples are randomly assigned a new group number, scrambling the sample group compositions, prior to performing the univariate analysis to determine the p-values for each peak position. By this method the number of peaks that is assigned a p-value below 0.01 by chance is determined. Iterative repetition of this procedure allows for a statistically relevant mean with standard deviation that could be taken as a realistic background value for this not normally distributed data.

The differentially abundant peptides were identified by nano-LC-ESI-Orbitrap MS. These measurements were carried out on a Ultimate 3000 nano LC system (Dionex, Germering, Germany) online coupled to a hybrid linear ion trap/Orbitrap MS (LTQ Orbitrap XL; Thermo Fisher Scientific, Germany). Five µL digest were loaded on to a C18 trap column (C18 PepMap, 300 µm ID ×5 mm, 5 µm particle size, 100 Å pore size; Dionex, The Netherlands) and desalted for 10 minutes using a flow rate of 20 µL/min 0.1% TFA. Then the trap column was switched online with the analytical column (PepMap C18, 75 µm ID ×150 mm, 3 µm particle and 100 Å pore size; Dionex, The Netherlands) and peptides were eluted with following binary gradient: 0%–25% solvent B in 120 min and 25%–50% solvent B in further 60 minutes, where solvent A consist of 2% acetonitrile and 0.1% formic in water and solvent B consists of 80% acetonitrile and 0.08% formic acid in water. Column flow rate was set to 300 nL/min. For MS detection a data dependent acquisition method was used: high resolution survey scan from 400–1800 Th. was performed in the Orbitrap (value of target of automatic gain control AGC 10^6^, resolution 30,000 at 400 m/z; lock mass was set to 445.120025 u (protonated (Si(CH_3_)_2_O)_6_) [15]). Based on this survey scan the 5 most intensive ions were consecutively isolated (AGC target set to 10^4^ ions) and fragmented by collision-activated dissociation (CAD) applying 35% normalized collision energy in the linear ion trap. After precursors were selected for MS/MS, they were excluded for further MS/MS spectra for 3 minutes. The MS/MS identifications were obtained using in the Bioworks 3.2 (peak picking by Extract_msn, default settings) software package (Thermo Fisher Scientific, Germany), and its' SEQUEST feature, using minimum XC scores of 1.8, 2.2 and 3.75 for reliable identification of single, double and triple charged ions respectively in the UniProt-database (version 56.0, human taxonomy (20069 entries)). Carboxymethylation of Cysteine (+57.021 u) as fixed and oxidation of Methionine (+15.996 u) as variable modifications and tryptic cleavage were considered. The number of allowed missed cleavages was 2, the mass tolerance for precursor ions was 10 ppm and for fragment ions 0.5 Da. The cut-off for mass differences with the theoretical mass of the identified peptides was set at 2 ppm.

Contamination of CSF by serum of plasma is a possible issue in CSF peptide profiling, because if one or more of the samples is contaminated by serum or plasma, the comparison of CSF peptide profiles is inevitably skewed by the higher total protein concentrations in serum or plasma [16]. To prevent inclusion of contaminated CSF samples in this study, the CSF samples were checked for specific blood contamination. If a hemoglobin peptide could be identified by nanoLC-ESI-Orbitrap (C_18_ column) with sufficiently high confidence score or if the mass peak 1274.7255 (part of hemoglobin gamma) has a signal to noise of 4 or higher in MALDI-FT-ICR measurements, the sample was discarded from further analysis due to plasma/serum contamination. Another blood specific protein, apolipoprotein B100 was checked in the same way as possible blood contamination.

### Immuno-assays for validation of differentially abundant proteins

For two proteins, for which we found differentially abundant peptides, we performed validation experiments. This was done by commercially available ELISA (for vitamin D-binding protein) and by western blot (for protein jagged-1), using a validation cohort of patients consisting of 10 RR MScl and 10 PP MScl samples. The samples of the validation cohort were demographically and clinically comparable to the original cohort of patients (average EDSS RR MScl group (standard deviation in brackets): 2.5 (0.8), average EDSS PP MScl group 2.8 (1.0), and the disease duration, presence/absence of oligoclonal IgG bands and male female ratio were all similar to the original cohort (no p-values below 0.05) using a t-test to compare the groups). Additionally we also measured the original samples using this ELISA and western blot. For the first protein, vitamin D-binding protein, we performed a commercially available ELISA (Immundiagnostik, Germany) according to the manufacturers specifications. For the second protein that was differentially abundant between the both MScl types, jagged-1, we performed a two-step western blot using goat anti-jagged1 antibodies (primary antibody) and anti-goat antibodies (secondary antibody) (Sigma Aldrich, United States). Protein transfer was checked by Ponceau staining. Quantitative assessment of the gel bands after photoluminescence was performed using Image J (freely available at rsb.info.nih.gov/ij).

## Results

### Clinical information

In total 34 CSF samples were used for mass spectrometry analysis, while twenty of these samples and twenty additional samples were used for validation experiments. All samples analyzed by mass spectrometry were tested as being negative for serum/plasma contamination by MALDI-FT-ICR measurements. However, in three samples we were able to identify hemoglobin peptides with sufficiently high XC scores for confident identification using nanoLC-ESI-Orbitrap measurements. These three samples, 2 PP MScl and 1 RR MScl, were subsequently excluded from further analysis. The analysis matrix, which was used to profile the differences in peptide profile between the three groups, consisted therefore of 31 CSF samples (Table 1 and Supplementary information Samples S1 file).

**Table 1:** CSF sample information.

	PP MScl	RR MScl	Controls
Number of samples	10	11	10
Protein concentration (g/L)	0.398 (0.118)	0.391 (0.135)	0.386 (0.110)
Albumin concentration (g/L)	0.254 (0.104)	0.228 (0.082)	0.205 (0.090)
Age	48.1 (9.0)	43.9 (14.1)	51.1 (13.7)
EDSS	3.2 (0.8)	2.8 (0.9)	-
Disease duration (years)	3.4 (1.3)	2.6 (1.5)	-
Male/Female ratio (% females in group)	6/4 (40%)	6/5 (45%)	8/2 (20%)

The concentrations, age, Expanded Disability Status Scale (EDSS) score and disease duration values are averages with standard deviation in brackets. None of the variables in these tables differed significantly between the groups (all t-tests showed p-values higher than 0.05).

### Peak detection and data analysis

After the MALDI-FT-ICR spectra were loaded into Peptrix™ software package, they were each tagged with a group number (1, 2 and 3 for PP MScl, RR MScl and controls respectively). Calibrating using five omnipresent albumin peaks was followed by generation of an analysis matrix with the intensity of all peaks of every sample recorded for all detected peaks (Supplementary Information Matrix S1 file). Using the Wilcoxon-Mann-Whitney test to compare the groups pair-wise, the comparison between both MScl types resulted in 15 peak masses with p-values below 0.01. By scrambling the sample groups the number of background peaks was determined at 17, so the number of differentially abundant peptide peaks in the comparison of the two MScl types is around the level of the number of background peaks, indicating that the difference between these two groups appears to be nonexistent or at least at background level. However, the proteins that were identified with low p-values in this comparison were of substantial interest in a MScl context.

A total of 43 peptide peaks with a p-value below 0.01 were observed for the comparison of PP MScl versus the controls. The comparison of RR MScl versus the controls had 41 peak masses with p-values lower than 0.01. Seventeen of the peak masses with p<0.01 were present in both comparisons.

### Identification

Identification of the differentially abundant peptides was performed by measuring all samples using the nanoLC-ESI-Orbitrap. Due to the prefractionating by nanoLC far more peak masses and identifications are generated by ESI-Orbitrap than there are peak masses in the analysis matrix generated by quantitative MALDI-FT-ICR. Although many of the identified peptides do not correspond to peak masses in the analysis matrix, we were able to identify a number of differentially abundant peptides for all three comparisons (full list, including charge states and sequence coverage, can be found in the supplementary material (Supplementary Information Mass Spectrometry S1 file)). Of the 43 differentially abundant peptide masses that were observed using MALDI-FT-ICR mass spectrometry in the comparison of PP MScl versus the controls we were able to identify 29 peptides (Supplementary Information Table S1). These peptides included several peptides of Ig gamma-1 and Ig kappa. Another differentially abundant peptide was identified as a part apolipoprotein D, which has previously been shown to be elevated intrathecally in MScl patients [17]. In the comparison of RR MScl versus controls we were able to identify 24 of the 41 differentially abundant peptide masses, which, as was the case with the comparison of PP MScl versus the controls, also included several peptides of Ig gamma-1 and Ig kappa as well as apolipoprotein D (Supplementary Information Table S2). In fact, of the 24 peptides identified in this comparison, 14 were also identified in the comparison of PP MScl versus the controls, indicating that the differences of the two MScl types compared to the controls are remarkably similar. Additional proteins with low p-values in the comparisons of both MScl types with the controls include StAR-related lipid transfer protein 4 (2.551 fold increase in PP MScl compared to controls and 2.837 fold increase in RR MScl compared to controls), LON peptidase N-terminal domain and RING finger protein (2.847 fold increase in PP MScl and 2.212 fold increase in RR MScl), and ryanodine receptor 1 (2.129 fold increase in PP MScl and 1.877 fold increase in RR MScl).

### PP MScl versus RR MScl

The comparison of PP MScl versus RR MScl showed a limited number of differentially abundant peptide peaks. Of these peaks 7 were identified, the most notable being protein jagged-1 (Table 2). This particular protein was over three times less abundant in PP MScl compared to RR MScl. Another interesting differentially abundant protein is vitamin D-binding protein, which was not detected by mass spectrometry in the PP MScl samples but was detected in the RR MScl samples. Other proteins, such as serine/threonine kinase NLK and sodium leak channel non-selective protein were more abundant in PP MScl than in RR MScl, although for the latter protein the difference was small (1.18 fold increase).

**Table 2:** Differentially abundant peptides and proteins in the comparison of PP MScl versus RR MScl.

Acc. number	Protein	# of pept.	p-value	Peptide	Abund. in PP MScl	Fold change	Incidence in PP MScl (%)	Incidence in RR MScl (%)
P02774	Vitamin D-binding protein	1	0.0092	ELPEHTVKLCDNLSTKNSK	↓	-	0	55
P61769	Beta-2-microglobulin	1	0.0014	VEHSDLSFSK	↑	-	70	0
P78504	Protein jagged-1	1	0.0087	TCMEGWM*GPECNRAICR	↓	3.188	30	63
Q8IZF0	Sodium leak channel non-selective protein	1	0.0069	GKSLETLTQDHSNTVRYR	↑	1.180	80	18
Q8NEB9	Phosphatidylinositol 3-kinase catalytic subunit type 3	1	0.0015	SALM*PAQLFFK	↓	-	0	73
Q9NXT0	Zinc finger protein568	1	0.0071	DQGGHSGERPYECGEYR	↓	1.786	80	82
Q9UBE8	Serine/threonine kinase NLK	1	0.0041	YHTCM*CKCCFSTSTGR	↑	-	60	0

M* denotes oxidation of methionine residue.

Because our main interest was focused on the differences between the two MScl types we selected two differentially abundant proteins from that comparison for validation purposes using 10 PP MScl and 10 RR MScl samples measured by mass spectrometry and an additional 10 PP MScl and 10 RR MScl samples from independent patients. Validation by ELISA showed that the concentration of vitamin D-binding protein was significantly lower (t-test, p<0.05) in the PP MScl group compared to the RR MScl group in the samples also measured by MALDI-FT-ICR MS (Table 3). A similar result was observed in the new, clinically and demographically comparable validation samples (Table 4) After western blotting, quantitative assessment of the gel bands showed that protein jagged-1 was indeed less abundant in PP MScl than in RR MScl. A t-test on the photoluminescence readout values of the original sample set showed a p-value below 0.05 when comparing the RR and PP MScl samples (Table 3). The same comparison in the validation samples also showed a p-value below 0.05), indicating a significant differential abundance of jagged-1 in the two MScl disease types (Table 4).

**Table 3:** The results of the validation experiments in the original sample set.

	PP MScl		RR MScl		
Original sample set	Average	Standard deviation	Average	Standard deviation	p-value (t-test)
Vitamin D-binding protein concentration in pg/ml (ELISA)	13716	5881	19594	3938	0.017
Protein jagged-1 photoluminescence readout (western blot)	8304	3553	16640	9563	0.019

By ELISA measurement vitamin D binding protein is more abundant in RR MScl than in PP MScl in the original sample set (p = 0.017), based on the average (+/− standard deviation) concentrations in CSF. Protein jagged-1 (western blot) is more abundant in RR MScl than in PP MScl in the original sample set (p = 0.019), based on the averages (+/− standard deviation) in photoluminescence readout.

**Table 4:** The results of the validation experiments in the validation sample set.

	PP MScl		RR MScl		
Validation sample set	Average	Standard deviation	Average	Standard deviation	p-value (t-test)
Vitamin D-binding protein concentration in pg/ml (ELISA)	15411	6186	23125	8458	0.032
Protein jagged-1 photoluminescence readout (western blot)	9462	3867	19868	14393	0.041

By ELISA measurement vitamin D binding protein is more abundant in RR MScl than in PP MScl in the demographically and clinically comparable validation sample set (p = 0.032), based on the average (+/− standard deviation) concentrations in CSF. Protein jagged-1 (western blot) is more abundant in RR MScl than in PP MScl in the validation sample set (p = 0.041), based on the averages (+/− standard deviation) in photoluminescence readout.

**Figure 1:**
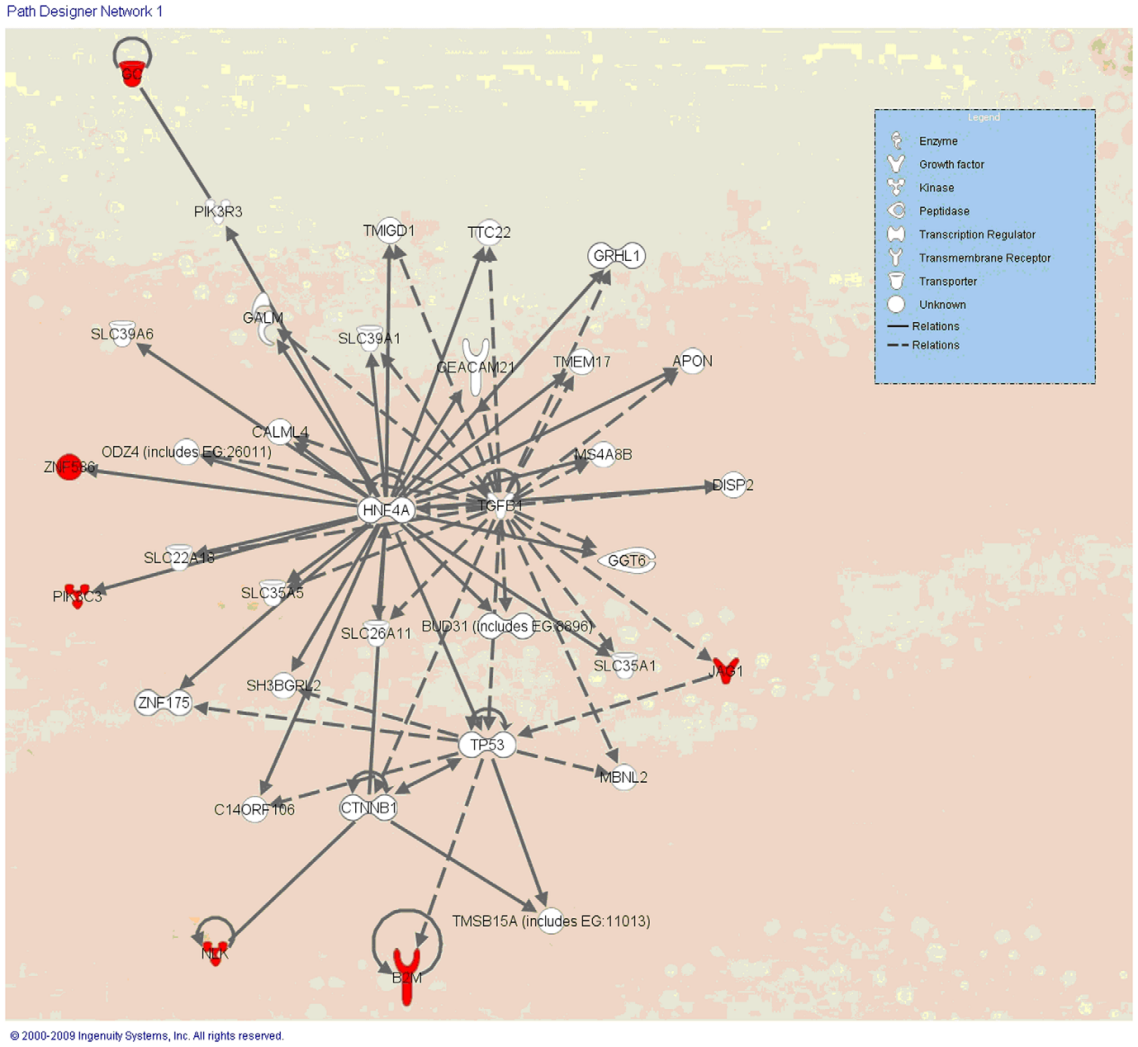
Six of the seven differentially abundant proteins (in red) identified in the comparison of the two MScl disease types (PP and RR) fit into a network related to neurological disease.

In order to place the identified proteins in a biological context, they were uploaded to the Ingenuity Pathways Analysis service (Ingenuity Systems) for to network analysis. Six of the seven differentially abundant proteins were placed in a network relating to neurological disease (Figure 1).

## Discussion

The main finding of this comparative study is the observation that the proteome profiles of CSF in PP vs RR MScl patients overlap to a large extent. This is in line with the lack of clear-cut differences between the two major clinical MScl sub-groups, at genetic, immunological and neuropathological levels. Interestingly, our approach using sensitive state-of the art mass spectrometry techniques, led to the identification of a few distinct CSF proteins, some of them with biological functions that appear of direct interest for MScl pathology. Two of these proteins were validated by other techniques as well as in a validation sample set. The lack of statistical power, due to the low number of biological samples available for this study, led to a less than ideal statistical analysis, meaning that the reported p-values may not be directly associated to disease pathology. However, the reported unvalidated proteins are of significant biological interest, and among these are several immunoglobulin proteins, which have been previously reported to be elevated in CSF of MScl patients [18], [19].

The number of peaks with low p-values in the comparison of the two MScl types is lower than the number of background peaks, so this is a strong indication that, even though there may be peptides and proteins that are differentially abundant in the comparison, overall the difference between the two disease types appears to be undetectable by means of the univariate analysis. One of the peptides of protein jagged-1 is over three times less abundant in PP MScl and also is observed with a lower incidence in this group (Table 2). Protein jagged-1 is a ligand for multiple Notch receptors and involved in the mediation of Notch signaling, which influences neuronal function and development [20]. The Notch signaling pathway has long been known to influence cell fate in the developing nervous system. Jagged-1 has been found to be highly expressed in hypertrophic astrocytes within and around active MScl plaques lacking remyelination, while, in contrast, there was negligible jagged-1 expression in remyelinated lesions suggesting involvement of the Notch pathway in remyelination in MScl [21]. Later, linkage equilibrium screening implicated a number of genes, including the jagged-1 gene, as susceptibility genes for MScl in a large contingent of Europeans [22]. It has also been suggested that jagged-1 has therapeutic potential in the treatment of CD8^+^ T cell mediated diseases, due to it's ability to deliver indirect negative signals into CD8^+^ T cells *in vivo* [23]. Additionally, animal models have shown that elevated expression of Notch and jagged-1 expression does not appear to be a limiting factor in remyelination, but the animal model study reports that there were no quantitative differences in Notch1 expressing cells in slow and rapidly remyelinating lesions, indicating that Notch–Jagged signaling is not a rate-limiting determinant of remyelination in rodent models of demyelination [24]. Additionally immunohistochemistry experiments have shown that constituents of the Notch pathway are expressed in remyelination in an animal model of T-cell- and antibody-mediated CNS demyelination [25]. However, network studies based on the quantitative expression levels of 20 genes in over one hundred individuals identify jagged-1 as a new therapeutic target whose differential behavior in the MScl network was not modified by immunomodulatory therapy, illustrating how network analysis can predict therapeutic targets for immune intervention and identifying the immunomodulatory properties of jagged-1, making it a new therapeutic target for MScl and other autoimmune diseases [26].

The identified peptide of vitamin D-binding protein is not observed in any of the PP MScl samples, but small peaks of this peptide are detected in 6/11 RR MScl samples. Impaired vitamin D homeostasis has been widely implicated in MScl for some years now [27]–[29]. This vitamin directly and indirectly regulates the differentiation, activation of CD4+ T-lymphocytes and can prevent the development of autoimmune processes [30], [31], and so it may be involved MScl. Considering that the geographic incidence of MScl indicates an increase in MScl with a decrease in sunlight exposure, that vitamin D is produced in the skin by solar or UV irradiation and that high serum levels of 25-hydroxyvitamin D have been reported to correlate with a reduced risk of MScl, a protective role of vitamin D has been suggested [32]. More than 99% of 25-hydroxyvitamin D, the principle circulating metabolite of vitamin D is bound to proteins, of which approximately 90% is bound to vitamin D-binding protein [33], [34]. Recently, a CSF proteomics study showed that serum levels of vitamin D-binding protein were decreased significantly in RR MScl patients compared to other neurological disorders[35]. While our results do not indicate a differential abundance difference between the MScl subtypes and the controls, the two disease types did vary significantly in CSF levels of vitamin D-binding protein, with RR MScl showing a higher abundance. Since a neuroprotective function has been suggested for vitamin D, it may be that in PP MScl this neuroprotective pathway is at least partially deficient, resulting in a significantly more disabling disease manifestation. However, it should not be forgotten that vitamin D-binding protein has pleiotropic functions, beyond vitamin D metabolism. It can significantly enhance the chemotactic response to complement fragment C5a [36], and there are substantial stimulatory effects on macrophages [37]. In light of the increasingly recognized role of innate immunity in the progressive phase of MS pathogenesis [38], vitamin D-binding protein appears an interesting candidate mediator.

The identification of a peptide of beta-2-microglobulin as differentially abundant in the comparison between both MScl disease types is somewhat misleading. Another seven peptides of this protein were identified among the peak masses in the analysis matrix that had high p-values in the comparison of PP and RR MScl, so it is very likely that the low p-value of the peptide of beta-2-microglobulin reflects high abundant protein variations, suggesting this low p-value is most likely a false positive. In contrast, the other proteins that are differentially abundant in this comparison were either identified by the single peptide listed in Table 2 or by multiple peptides that had low p-values.

The identification of peptides of albumin and serotransferrin stands out in the tables of the differentially abundant peptides and proteins. While the identifications are essentially correct it must be noted that only 2.4% of the identified albumin peptides and 4.8% of the identified serotransferrin peptides had p-values below 0.01, which means that the values for the peptides of these two particular proteins are most likely due to other reasons than large abundance differences in these two proteins. In comparison, most of the other differentially abundant proteins that we identified were found by a small number of peptides with a low p-value or a single peptide with a low p-value. For these proteins we did not observe any other peptides with non-significant p-values, with the exception of bromodomain adjacent to zinc finger domain protein 1A and Rho GTPase-activating protein 18-like. For both of these proteins another peptide was identified with high p-value, indicating these proteins were likely not differentially abundant. The peptides with low p-values of these proteins were also characterized by a low fold increase, making them less interesting for independent immunoassay follow-up.

Because of the healthy state of the CSF control group, no intrathecal inflammatory response was to be expected in this group. Therefore the very clear difference in immunoglobulin abundance in the comparison with the both MScl disease types can be explained. In the two comparisons of both MScl types with the control group, several proteins are present in both comparisons, for example Ig gamma-1 chain C region and apolipoprotein D. Apolipoproteins have been previously implicated in MScl. Proteomics studies have shown apolipoprotein E abundances to be elevated in CSF of MScl patients compared to controls [9], [12]. In the central nervous system apolipoprotein D is a lipocalin that is mainly expressed in glia, but also in neurons. This protein has been repeatedly implicated in MScl, and it has been shown that it has a neuroprotective effect in a number of neurodegenerative diseases by controlling the level of peroxidated lipids, which coincides with glial activation in mouse models of encephalitis [39]. A previous proteomics study showed increased levels of apolipoprotein D in patients with a clinically isolated syndrome of demyelination, indicating that abundance levels of this protein are highest in MScl patients at the time of their first exacerbation [10], [17]. Another potentially interesting protein found to be differentially elevated in the comparisons of both MScl types with the controls is ryanodine receptor 1. This receptor is involved in the maintenance of the calcium-equilibrium in brain tissue. The release of toxic levels of positively charged calcium ions may, due to the deleterious effects of excitotoxicity, represent a key mechanism of axonal degeneration in disorders such as MScl [40].

In conclusion, the CSF peptide profile of the control samples differed from both MScl types, with, not unexpectedly, proteins related to immune response showing the highest fold increase in abundance in the MScl types compared to the controls. Even though the CSF peptide profiles measured by MALDI-FT-ICR of PP MScl and RR MScl were quite similar, still a few differences could be observed, most notably regarding the molecules confirmed by immunoassay, protein jagged-1 and vitamin D-binding protein.

## Supporting Information

Table S1 (0.07 MB DOC)

Table S2 (0.07 MB DOC)

Samples S1 (0.03 MB XLS)

Matrix S1 (0.96 MB XLSX)

Mass Spectrometry S1 (0.04 MB XLS)

## References

[1] Compston A, Coles A (2008). Multiple sclerosis.. Lancet.

[2] Miller DH, Leary SM (2007). Primary-progressive multiple sclerosis.. Lancet Neurol.

[3] Hoppenbrouwers IA, Cortes LM, Aulchenko YS, Sintnicolaas K, Njajou O (2007). Familial clustering of multiple sclerosis in a Dutch genetic isolate.. Mult Scler.

[4] Richards RG, Sampson FC, Beard SM, Tappenden P (2002). A review of the natural history and epidemiology of multiple sclerosis: implications for resource allocation and health economic models.. Health Technol Assess.

[5] Lassmann H (2007). New concepts on progressive multiple sclerosis.. Curr Neurol Neurosci Rep.

[6] Han MH, Hwang SI, Roy DB, Lundgren DH, Price JV (2008). Proteomic analysis of active multiple sclerosis lesions reveals therapeutic targets.. Nature.

[7] Liu S, Bai S, Qin Z, Yang Y, Cui Y (2009). Quantitative proteomic analysis of the cerebrospinal fluid of patients with multiple sclerosis.. J Cell Mol Med.

[8] Tumani H, Lehmensiek V, Rau D, Guttmann I, Tauscher G (2009). CSF proteome analysis in clinically isolated syndrome (CIS): candidate markers for conversion to definite multiple sclerosis.. Neurosci Lett.

[9] Lehmensiek V, Sussmuth SD, Tauscher G, Brettschneider J, Felk S (2007). Cerebrospinal fluid proteome profile in multiple sclerosis.. Mult Scler.

[10] Stoop MP, Dekker LJ, Titulaer MK, Burgers PC, Sillevis Smitt PA (2008). Multiple sclerosis-related proteins identified in cerebrospinal fluid by advanced mass spectrometry.. Proteomics.

[11] Chiasserini D, Di Filippo M, Candeliere A, Susta F, Orvietani PL (2008). CSF proteome analysis in multiple sclerosis patients by two-dimensional electrophoresis.. Eur J Neurol.

[12] Stoop MP, Dekker LJ, Titulaer MK, Lamers RJ, Burgers PC (2009). Quantitative matrix-assisted laser desorption ionization-fourier transform ion cyclotron resonance (MALDI-FT-ICR) peptide profiling and identification of multiple-sclerosis-related proteins.. J Proteome Res.

[13] McDonald WI, Compston A, Edan G, Goodkin D, Hartung HP (2001). Recommended diagnostic criteria for multiple sclerosis: guidelines from the International Panel on the diagnosis of multiple sclerosis.. Ann Neurol.

[14] Montalban X, Sastre-Garriga J, Filippi M, Khaleeli Z, Tellez N (2009). Primary progressive multiple sclerosis diagnostic criteria: a reappraisal.. Mult Scler.

[15] Olsen JV, de Godoy LM, Li G, Macek B, Mortensen P (2005). Parts per million mass accuracy on an Orbitrap mass spectrometer via lock mass injection into a C-trap.. Mol Cell Proteomics.

[16] You JS, Gelfanova V, Knierman MD, Witzmann FA, Wang M (2005). The impact of blood contamination on the proteome of cerebrospinal fluid.. Proteomics.

[17] Reindl M, Knipping G, Wicher I, Dilitz E, Egg R (2001). Increased intrathecal production of apolipoprotein D in multiple sclerosis.. J Neuroimmunol.

[18] Di Pauli F, Gredler V, Kuenz B, Lutterotti A, Ehling R (2010). Features of intrathecal immunoglobulins in patients with multiple sclerosis.. J Neurol Sci.

[19] Presslauer S, Milosavljevic D, Brucke T, Bayer P, Hubl W (2008). Elevated levels of kappa free light chains in CSF support the diagnosis of multiple sclerosis.. J Neurol.

[20] Gaiano N, Fishell G (2002). The role of notch in promoting glial and neural stem cell fates.. Annu Rev Neurosci.

[21] John GR, Shankar SL, Shafit-Zagardo B, Massimi A, Lee SC (2002). Multiple sclerosis: re-expression of a developmental pathway that restricts oligodendrocyte maturation.. Nat Med.

[22] The Games Collaborative G, Ban M, Booth D, Heard R, Stewart G (2006). Linkage disequilibrium screening for multiple sclerosis implicates JAG1 and POU2AF1 as susceptibility genes in Europeans.. J Neuroimmunol.

[23] Kijima M, Iwata A, Maekawa Y, Uehara H, Izumi K (2009). Jagged1 suppresses collagen-induced arthritis by indirectly providing a negative signal in CD8+ T cells.. J Immunol.

[24] Stidworthy MF, Genoud S, Li WW, Leone DP, Mantei N (2004). Notch1 and Jagged1 are expressed after CNS demyelination, but are not a major rate-determining factor during remyelination.. Brain.

[25] Seifert T, Bauer J, Weissert R, Fazekas F, Storch MK (2007). Notch1 and its ligand Jagged1 are present in remyelination in a T-cell- and antibody-mediated model of inflammatory demyelination.. Acta Neuropathol.

[26] Palacios R, Goni J, Martinez-Forero I, Iranzo J, Sepulcre J (2007). A network analysis of the human T-cell activation gene network identifies JAGGED1 as a therapeutic target for autoimmune diseases.. PLoS ONE.

[27] Cantorna MT (2000). Vitamin D and autoimmunity: is vitamin D status an environmental factor affecting autoimmune disease prevalence?. Proc Soc Exp Biol Med.

[28] Lips P (2004). Which circulating level of 25-hydroxyvitamin D is appropriate?. J Steroid Biochem Mol Biol.

[29] Szodoray P, Nakken B, Gaal J, Jonsson R, Szegedi A (2008). The complex role of vitamin D in autoimmune diseases.. Scand J Immunol.

[30] Adorini L (2001). Selective immunointervention in autoimmune diseases: lessons from multiple sclerosis.. J Chemother.

[31] Cantorna MT, Mahon BD (2004). Mounting evidence for vitamin D as an environmental factor affecting autoimmune disease prevalence.. Exp Biol Med (Maywood).

[32] Raghuwanshi A, Joshi SS, Christakos S (2008). Vitamin D and multiple sclerosis.. J Cell Biochem.

[33] Bikle DD, Siiteri PK, Ryzen E, Haddad JG (1985). Serum protein binding of 1,25-dihydroxyvitamin D: a reevaluation by direct measurement of free metabolite levels.. J Clin Endocrinol Metab.

[34] Bikle DD, Gee E, Halloran B, Kowalski MA, Ryzen E (1986). Assessment of the free fraction of 25-hydroxyvitamin D in serum and its regulation by albumin and the vitamin D-binding protein.. J Clin Endocrinol Metab.

[35] Qin Z, Qin Y, Liu S (2009). Alteration of DBP levels in CSF of patients with MS by proteomics analysis.. Cell Mol Neurobiol.

[36] Shah AB, DiMartino SJ, Trujillo G, Kew RR (2006). Selective inhibition of the C5a chemotactic cofactor function of the vitamin D binding protein by 1,25(OH)2 vitamin D3.. Mol Immunol.

[37] Gomme PT, Bertolini J (2004). Therapeutic potential of vitamin D-binding protein.. Trends Biotechnol.

[38] Weiner HL (2009). The challenge of multiple sclerosis: how do we cure a chronic heterogeneous disease?. Ann Neurol.

[39] Do Carmo S, Jacomy H, Talbot PJ, Rassart E (2008). Neuroprotective effect of apolipoprotein D against human coronavirus OC43-induced encephalitis in mice.. J Neurosci.

[40] Ouardouz M, Coderre E, Basak A, Chen A, Zamponi GW (2009). Glutamate receptors on myelinated spinal cord axons: I. GluR6 kainate receptors.. Ann Neurol.

