# Supplementary material for: Proteomics Comparison of Cerebrospinal Fluid of Relapsing Remitting and Primary Progressive Multiple Sclerosis
Source: PLoS One. 2010 Aug 27;5(8):e12442. doi: 10.1371/journal.pone.0012442 (PMC2929207; doi:10.1371/journal.pone.0012442)
Supplement: Table S1 — (0.07 MB DOC) [file pone.0012442.s001.doc]

Supplementary Information Table S1: Differentially abundant peptides and proteins in the comparison of PP MScl versus controls.

| Acc. number | Protein | # of pept. | p-value | Peptide | Abund. in PP MScl | Fold change | Incidence in  PP MScl (%) | Incidence in  Controls (%) |
| --- | --- | --- | --- | --- | --- | --- | --- | --- |
| A1L0T0 | Acetolactate synthase-like protein | 1 | 0.0038 | ENEDQVVKVLHDAQQQCR | ↑ | 1.891 | 80 | 30 |
| O43707 | Alpha-actinin-4 | 1 | 0.0032 | M*APYQGPDAVPGALDYK | ↑ | 1.998 | 70 | 10 |
| O43166 | Signal-induced proliferation-associated 1-like protein 1 | 1 | 0.0036 | SQNGSLGSSVMAPVGPPR | ↑ | 1.540 | 80 | 20 |
| O75746 | Calcium-binding mitochondrial carrier protein Aralar1 | 1 | 0.0092 | EEGPSAFWKGTAARVFR | ↑ | 1.513 | 40 | 50 |
| P01614 | Ig kappa chain V-II region Cum | 1 | 0.0069 | LEIPYTFGQGTKLEIR | ↑ | 1.376 | 70 | 10 |
| P01834 | Ig kappa chain C region | 2 | 0.0035 | SGTASVVCLNNFYPR | ↑ | 2.761 | 100 | 100 |
| P01834 | Ig kappa chain C region | 2 | 0.0054 | TVAAPSVFIFPPSDEQLK | ↑ | 2.478 | 100 | 80 |
| P01857 | Ig gamma-1 chain C region | 3 | 0.0008 | VVSVLTVLHQDWLNGK | ↑ | 3.036 | 100 | 90 |
| P01857 | Ig gamma-1 chain C region | 3 | 0.0011 | FNWYVDGVEVHNAK | ↑ | 4.048 | 100 | 100 |
| P01857 | Ig gamma-1 chain C region | 3 | 0.0054 | TPEVTCVVVDVSHEDPEVK | ↑ | 2.392 | 100 | 80 |
| P02768 | Albumin | 1 | 0.0069 | DVFLGM*FLYEYAR | ↑ | 1.732 | 100 | 80 |
| P02787 | Serotransferrin | 1 | 0.0032 | SMGGKEDLIWELLNQAQEHFGK | ↓ | 1.301 | 20 | 80 |
| P21817 | Ryanodine receptor 1 | 1 | 0.0064 | EIRFPKMVTSCCR | ↑ | 2.129 | 70 | 10 |
| P05090 | Apolipoprotein D | 1 | 0.0054 | NPNLPPETVDSLK | ↑ | 2.579 | 100 | 50 |
| Q1L5Z9 | LON peptidase N-terminal domain and RING finger protein | 1 | 0.0044 | TFPDGSSVVDAIGISRFRVLSHR | ↑ | 2.847 | 50 | 20 |
| Q2NKQ1 | Small G protein signaling modulator 1 | 1 | 0.0017 | NTPTVLRPRDGSVDDR | ↑ | 2.259 | 100 | 70 |
| Q5TG30 | Rho GTPase-activating protein 18-like | 1 | 0.0082 | IGDLSLQDM*R | ↓ | 1.124 | 20 | 80 |
| Q7L3B6 | Hsp90 co-chaperone Cdc37-like 1 | 1 | 0.0069 | MCLWSTDAISKDVFNKSFINQDK | ↑ | 1.719 | 80 | 20 |
| Q8IZF0 | Sodium leak channel non-selective protein | 1 | 0.0034 | GKSLETLTQDHSNTVRYR | ↑ | 1.625 | 80 | 20 |
| Q8N8H1 | KRAB domain-containing protein ZNF321 | 1 | 0.0032 | HESHHHIRDFCFQEIEK | ↓ | 1.577 | 30 | 90 |
| Q8NEB9 | Phosphatidylinositol 3-kinase catalytic subunit type 3 | 1 | 0.0092 | SALM*PAQLFFK | ↓ | - | 0 | 60 |
| Q96DR4 | StAR-related lipid transfer protein 4 | 1 | 0.0044 | GYNHPCGWFCVPLK | ↑ | 2.551 | 70 | 10 |
| Q96RD6 | Pannexin-2 | 1 | 0.0038 | GGGGDPGPGPAPAPAPPPAPDK | ↓ | 1.458 | 10 | 70 |
| Q9BZ29 | Dedicator of cytokinesis protein 9 | 1 | 0.0063 | YAYKAEPYVASEYK | ↑ | 2.794 | 70 | 20 |
| Q9NRL2 | Bromodomain adjacent to zinc finger domain protein 1A | 1 | 0.0071 | QEQINCVTR | ↓ | 1.039 | 30 | 100 |
| Q9NW82 | WD repeat-containing protein 70 | 1 | 0.0042 | KVIPTTCTYSR | ↑ | 2.574 | 70 | 50 |
| Q9PKX4 | Docking protein 6 | 1 | 0.0054 | M*CDTGEGLFTFQTRREGEMIYQK | ↑ | 1.191 | 70 | 10 |
| Q9UPA5 | Protein bassoon | 1 | 0.0054 | HSYHDYDEPPEEGLWPHDEGGPGRHASAK | ↑ | 1.391 | 90 | 10 |
| Q9Y2P8 | RNA 3’-terminal phosphate cyclase-like protein | 1 | 0.0063 | GMAYSVRVSPQM*ANR | ↑ | 1.922 | 70 | 10 |

M* denotes oxidation of methionine residue.
