# Supplementary material for: Proteomics Comparison of Cerebrospinal Fluid of Relapsing Remitting and Primary Progressive Multiple Sclerosis
Source: PLoS One. 2010 Aug 27;5(8):e12442. doi: 10.1371/journal.pone.0012442 (PMC2929207; doi:10.1371/journal.pone.0012442)
Supplement: Table S2 — (0.07 MB DOC) [file pone.0012442.s002.doc]

Supplementary Information Table S2: Differentially abundant peptides and proteins in the comparison of RR MScl versus controls.

| Acc. number | Protein | # of pept. | p-value | Peptide | Abund. in RR MScl | Fold change | Incidence in  RR MScl (%) | Incidence in  Controls (%) |
| --- | --- | --- | --- | --- | --- | --- | --- | --- |
| O15033 | Protein KIAA0317 | 1 | 0.0070 | EFYLKIIPWRLYTFR | ↑ | 1.445 | 100 | 80 |
| O43166 | Signal-induced proliferation-associated 1-like protein 1 | 1 | 0.0043 | SQNGSLGSSVMAPVGPPR | ↑ | 1.482 | 64 | 20 |
| P01009 | Alpha-1-antitrypsin | 1 | 0.0086 | GKWERPFEVK | ↑ | 1.444 | 100 | 100 |
| P01614 | Ig kappa chain V-II region Cum | 1 | 0.0032 | LEIPYTFGQGTKLEIR | ↑ | 1.678 | 73 | 10 |
| P01834 | Ig kappa chain C region | 2 | 0.0016 | SGTASVVCLNNFYPR | ↑ | 2.790 | 100 | 80 |
| P01834 | Ig kappa chain C region | 2 | 0.0047 | TVAAPSVFIFPPSDEQLK | ↑ | 2.967 | 100 | 100 |
| P01857 | Ig gamma-1 chain C region | 4 | 0.0038 | VVSVLTVLHQDWLNGK | ↑ | 2.418 | 100 | 70 |
| P01857 | Ig gamma-1 chain C region | 4 | 0.0047 | GPSVFPLAPSSK | ↑ | 3.872 | 100 | 100 |
| P01857 | Ig gamma-1 chain C region | 4 | 0.0058 | TPEVTCVVVDVSHEDPEVK | ↑ | 2.698 | 100 | 80 |
| P01857 | Ig gamma-1 chain C region | 4 | 0.0071 | FNWYVDGVEVHNAK | ↑ | 3.804 | 91 | 90 |
| P05090 | Apolipoprotein D | 1 | 0.0051 | NPNLPPETVDSLK | ↑ | 2.824 | 82 | 50 |
| P07093 | Glia-derived nexin | 1 | 0.0058 | VLGITDM*FDSSKANFAK | ↑ | 2.097 | 73 | 10 |
| P12931 | Proto-oncogene tyrosine-protein kinase Src | 1 | 0.0065 | TQFNSLQQLVAYYSKHADGLCHR | ↑ | 1.329 | 73 | 10 |
| P21817 | Ryanodine receptor 1 | 1 | 0.0063 | EIRFPKMVTSCCR | ↑ | 1.877 | 55 | 10 |
| Q12772 | Sterol regulatory element-binding protein 2 | 1 | 0.0055 | LPAGSACSDVHMALCAVNLAECAEEK | ↑ | 1.284 | 64 | 80 |
| Q13555 | Calcium/calmodulin-dependent protein kinase type II gamma chain | 1 | 0.0047 | KTSTQEYAAK | ↓ | 1.413 | 27 | 80 |
| Q19AV6 | Zinc finger SWIM domain-containing protein 7 | 1 | 0.0065 | HLLAVYLSQVMRTCQQSVSDk | ↓ | 1.318 | 36 | 20 |
| Q1L5Z9 | LON peptidase N-terminal domain and RING finger protein | 1 | 0.0058 | TFPDGSSVVDAIGISRFRVLSHR | ↑ | 2.212 | 73 | 20 |
| Q2NKQ1 | Small G protein signaling modulator 1 | 1 | 0.0016 | NTPTVLRPRDGSVDDR | ↑ | 2.417 | 100 | 70 |
| Q86VR8 | Four-jointed box protein 1 | 1 | 0.0065 | SEPRWHVSARQPR | ↓ | - | 0 | 60 |
| Q96DR4 | StAR-related lipid transfer protein 4 | 1 | 0.0032 | GYNHPCGWFCVPLK | ↑ | 2.837 | 27 | 10 |
| Q9NW82 | WD repeat-containing protein 70 | 1 | 0.0041 | KVIPTTCTYSR | ↑ | 3.054 | 82 | 50 |
| Q9NXT0 | Zinc finger protein568 | 1 | 0.0062 | DQGGHSGERPYECGEYR | ↑ | 1.969 | 82 | 30 |
| Q9Y2P8 | RNA 3’-terminal phosphate cyclase-like protein | 1 | 0.0060 | GMAYSVRVSPQM*ANR | ↑ | 2.112 | 64 | 10 |

M* denotes oxidation of methionine residue.
